# Supplementary material for: The contribution of abortive infection to preventing populations of Lactococcus lactis from succumbing to infections with bacteriophage
Source: PLoS One. 2024 Apr 1;19(4):e0298680. doi: 10.1371/journal.pone.0298680 (PMC10984412; doi:10.1371/journal.pone.0298680)
Supplement: Attachment — Submitted filename: response to reviewers Abi12-14.docx [file pone.0298680.s002.docx]

**From:** [em.pone.0.8756ff.2dbe9707@editorialmanager.com](mailto:em.pone.0.8756ff.2dbe9707@editorialmanager.com) <[em.pone.0.8756ff.2dbe9707@editorialmanager.com](mailto:em.pone.0.8756ff.2dbe9707@editorialmanager.com)> on behalf of PLOS ONE <[em@editorialmanager.com](mailto:em@editorialmanager.com)>
**Date:** Sunday, November 12, 2023 at 8:41 PM
**To:** Levin, Bruce <[blevin@emory.edu](mailto:blevin@emory.edu)>
**Subject:** [External] PLOS ONE Decision: Revision required [PONE-D-23-26559] - [EMID:44149bfb51625897]

PONE-D-23-26559

The contribution of abortive infection to preventing populations of Lactococcus lactis from succumbing to infections with bacteriophage

PLOS ONE

Dear Dr. Levin,

Thank you for submitting your manuscript to PLOS ONE. After careful consideration, we feel that it has merit but does not fully meet PLOS ONE’s publication criteria as it currently stands. Therefore, we invite you to submit a revised version of the manuscript that addresses the points raised during the review process.

Please submit your revised manuscript by Dec 27 2023 11:59PM. If you will need more time than this to complete your revisions, please reply to this message or contact the journal office at [plosone@plos.org](mailto:plosone@plos.org). When you're ready to submit your revision, log on to [https://www.editorialmanager.com/pone/](https://nam11.safelinks.protection.outlook.com/?url=https%3A%2F%2Fwww.editorialmanager.com%2Fpone%2F&data=05%7C01%7Cblevin%40emory.edu%7C5189c916b524474ad24108dbe47aaa4c%7Ce004fb9cb0a4424fbcd0322606d5df38%7C0%7C0%7C638354989737656019%7CUnknown%7CTWFpbGZsb3d8eyJWIjoiMC4wLjAwMDAiLCJQIjoiV2luMzIiLCJBTiI6Ik1haWwiLCJXVCI6Mn0%3D%7C3000%7C%7C%7C&sdata=aNtb1O6ByhrrPvGmpxtC6otif%2F3KiBU9jtritStbD6c%3D&reserved=0) and select the 'Submissions Needing Revision' folder to locate your manuscript file.

Please include the following items when submitting your revised manuscript:

- A rebuttal letter that responds to each point raised by the academic editor and reviewer(s). You should upload this letter as a separate file labeled 'Response to Reviewers'.
- A marked-up copy of your manuscript that highlights changes made to the original version. You should upload this as a separate file labeled 'Revised Manuscript with Track Changes'.
- An unmarked version of your revised paper without tracked changes. You should upload this as a separate file labeled 'Manuscript'.

If you would like to make changes to your financial disclosure, please include your updated statement in your cover letter. Guidelines for resubmitting your figure files are available below the reviewer comments at the end of this letter.

If applicable, we recommend that you deposit your laboratory protocols in [protocols.io](https://nam11.safelinks.protection.outlook.com/?url=http%3A%2F%2Fprotocols.io%2F&data=05%7C01%7Cblevin%40emory.edu%7C5189c916b524474ad24108dbe47aaa4c%7Ce004fb9cb0a4424fbcd0322606d5df38%7C0%7C0%7C638354989737656019%7CUnknown%7CTWFpbGZsb3d8eyJWIjoiMC4wLjAwMDAiLCJQIjoiV2luMzIiLCJBTiI6Ik1haWwiLCJXVCI6Mn0%3D%7C3000%7C%7C%7C&sdata=tUXgk%2B402C%2B%2B71UfQvC7K1y3fF4CA9hVT7k%2F5If5oCs%3D&reserved=0) to enhance the reproducibility of your results. Protocols.io assigns your protocol its own identifier (DOI) so that it can be cited independently in the future. For instructions see: [https://journals.plos.org/plosone/s/submission-guidelines#loc-laboratory-protocols](https://nam11.safelinks.protection.outlook.com/?url=https%3A%2F%2Fjournals.plos.org%2Fplosone%2Fs%2Fsubmission-guidelines%23loc-laboratory-protocols&data=05%7C01%7Cblevin%40emory.edu%7C5189c916b524474ad24108dbe47aaa4c%7Ce004fb9cb0a4424fbcd0322606d5df38%7C0%7C0%7C638354989737656019%7CUnknown%7CTWFpbGZsb3d8eyJWIjoiMC4wLjAwMDAiLCJQIjoiV2luMzIiLCJBTiI6Ik1haWwiLCJXVCI6Mn0%3D%7C3000%7C%7C%7C&sdata=uumKpQr3fPre%2B4GMuCkHDFxkqR5JW2NTwFvoxoHuIR8%3D&reserved=0). Additionally, PLOS ONE offers an option for publishing peer-reviewed Lab Protocol articles, which describe protocols hosted on [protocols.io](https://nam11.safelinks.protection.outlook.com/?url=http%3A%2F%2Fprotocols.io%2F&data=05%7C01%7Cblevin%40emory.edu%7C5189c916b524474ad24108dbe47aaa4c%7Ce004fb9cb0a4424fbcd0322606d5df38%7C0%7C0%7C638354989737656019%7CUnknown%7CTWFpbGZsb3d8eyJWIjoiMC4wLjAwMDAiLCJQIjoiV2luMzIiLCJBTiI6Ik1haWwiLCJXVCI6Mn0%3D%7C3000%7C%7C%7C&sdata=tUXgk%2B402C%2B%2B71UfQvC7K1y3fF4CA9hVT7k%2F5If5oCs%3D&reserved=0). Read more information on sharing protocols at [https://plos.org/protocols?utm_medium=editorial-email&utm_source=authorletters&utm_campaign=protocols](https://nam11.safelinks.protection.outlook.com/?url=https%3A%2F%2Fplos.org%2Fprotocols%3Futm_medium%3Deditorial-email%26utm_source%3Dauthorletters%26utm_campaign%3Dprotocols&data=05%7C01%7Cblevin%40emory.edu%7C5189c916b524474ad24108dbe47aaa4c%7Ce004fb9cb0a4424fbcd0322606d5df38%7C0%7C0%7C638354989737656019%7CUnknown%7CTWFpbGZsb3d8eyJWIjoiMC4wLjAwMDAiLCJQIjoiV2luMzIiLCJBTiI6Ik1haWwiLCJXVCI6Mn0%3D%7C3000%7C%7C%7C&sdata=zbvMsp6uTM6ogSS%2F1YiPAgPGEAdEyvVg4af6PiQRblQ%3D&reserved=0).

We look forward to receiving your revised manuscript.

Kind regards,

Konstantinos Papadimitriou, Ph.D.
Academic Editor
PLOS ONE

Journal Requirements:

When submitting your revision, we need you to address these additional requirements.

1. Please ensure that your manuscript meets PLOS ONE's style requirements, including those for file naming. The PLOS ONE style templates can be found at 
[https://journals.plos.org/plosone/s/file?id=wjVg/PLOSOne_formatting_sample_main_body.pdf](https://nam11.safelinks.protection.outlook.com/?url=https%3A%2F%2Fjournals.plos.org%2Fplosone%2Fs%2Ffile%3Fid%3DwjVg%2FPLOSOne_formatting_sample_main_body.pdf&data=05%7C01%7Cblevin%40emory.edu%7C5189c916b524474ad24108dbe47aaa4c%7Ce004fb9cb0a4424fbcd0322606d5df38%7C0%7C0%7C638354989737812245%7CUnknown%7CTWFpbGZsb3d8eyJWIjoiMC4wLjAwMDAiLCJQIjoiV2luMzIiLCJBTiI6Ik1haWwiLCJXVCI6Mn0%3D%7C3000%7C%7C%7C&sdata=7grWdzdWtMdVjqbMY9n%2FQbbBktEwzgDpank1%2Bjm%2BBEw%3D&reserved=0) and 
[https://journals.plos.org/plosone/s/file?id=ba62/PLOSOne_formatting_sample_title_authors_affiliations.pdf](https://nam11.safelinks.protection.outlook.com/?url=https%3A%2F%2Fjournals.plos.org%2Fplosone%2Fs%2Ffile%3Fid%3Dba62%2FPLOSOne_formatting_sample_title_authors_affiliations.pdf&data=05%7C01%7Cblevin%40emory.edu%7C5189c916b524474ad24108dbe47aaa4c%7Ce004fb9cb0a4424fbcd0322606d5df38%7C0%7C0%7C638354989737812245%7CUnknown%7CTWFpbGZsb3d8eyJWIjoiMC4wLjAwMDAiLCJQIjoiV2luMzIiLCJBTiI6Ik1haWwiLCJXVCI6Mn0%3D%7C3000%7C%7C%7C&sdata=phxMHFyFx8ullGP9R9O%2FQ8F5Mh298IX9EotjGku36HU%3D&reserved=0)

2. Please note that PLOS ONE has specific guidelines on code sharing for submissions in which author-generated code underpins the findings in the manuscript. In these cases, all author-generated code must be made available without restrictions upon publication of the work. Please review our guidelines at [https://journals.plos.org/plosone/s/materials-and-software-sharing#loc-sharing-code](https://nam11.safelinks.protection.outlook.com/?url=https%3A%2F%2Fjournals.plos.org%2Fplosone%2Fs%2Fmaterials-and-software-sharing%23loc-sharing-code&data=05%7C01%7Cblevin%40emory.edu%7C5189c916b524474ad24108dbe47aaa4c%7Ce004fb9cb0a4424fbcd0322606d5df38%7C0%7C0%7C638354989737812245%7CUnknown%7CTWFpbGZsb3d8eyJWIjoiMC4wLjAwMDAiLCJQIjoiV2luMzIiLCJBTiI6Ik1haWwiLCJXVCI6Mn0%3D%7C3000%7C%7C%7C&sdata=oIJCjNxhPabbcAzuX71StfFF%2BBL4bvZL1ZqXHkLjeyk%3D&reserved=0) and ensure that your code is shared in a way that follows best practice and facilitates reproducibility and reuse.

3. We note you have included a table to which you do not refer in the text of your manuscript. Please ensure that you refer to Table 2 in your text; if accepted, production will need this reference to link the reader to the Table.

We added a reference to Table 2. We thank the editor for this comment.

4. Please include captions for your Supporting Information files at the end of your manuscript, and update any in-text citations to match accordingly. Please see our Supporting Information guidelines for more information: [http://journals.plos.org/plosone/s/supporting-information](https://nam11.safelinks.protection.outlook.com/?url=http%3A%2F%2Fjournals.plos.org%2Fplosone%2Fs%2Fsupporting-information&data=05%7C01%7Cblevin%40emory.edu%7C5189c916b524474ad24108dbe47aaa4c%7Ce004fb9cb0a4424fbcd0322606d5df38%7C0%7C0%7C638354989737812245%7CUnknown%7CTWFpbGZsb3d8eyJWIjoiMC4wLjAwMDAiLCJQIjoiV2luMzIiLCJBTiI6Ik1haWwiLCJXVCI6Mn0%3D%7C3000%7C%7C%7C&sdata=INAX4idikGNh3m2lJOdH38aTr6xDnTmoKd8ElANJU3o%3D&reserved=0).

We added these captions after the References.

Additional Editor Comments:
Dear Authors, I believe that your study is interesting and of importance. However some major revisions are required before the study can be considered for publication.

Dear editor. We thank you for considering this manuscript interesting and important. Obviously, we agree.

[Note: HTML markup is below. Please do not edit.]

Reviewers' comments:

Reviewer's Responses to Questions

**Comments to the Author**

1. Is the manuscript technically sound, and do the data support the conclusions?

The manuscript must describe a technically sound piece of scientific research with data that supports the conclusions. Experiments must have been conducted rigorously, with appropriate controls, replication, and sample sizes. The conclusions must be drawn appropriately based on the data presented.

Reviewer #1: Yes

Reviewer #2: Partly

We appreciate this reviewer carefully working through this manuscript. It’s not clear why this reviewer is only “partly” sure this about this manuscript being technically sound and the data supporting the conclusions. More information would be helpful.

Reviewer #3: Yes

2. Has the statistical analysis been performed appropriately and rigorously?

Reviewer #1: Yes

Reviewer #2: I Don't Know

We don’t know why this reviewer questions whether our statistical analysis been performed appropriately and rigorously. More information would be helpful.

Reviewer #3: Yes

3. Have the authors made all data underlying the findings in their manuscript fully available?

The [PLOS Data policy](https://nam11.safelinks.protection.outlook.com/?url=http%3A%2F%2Fwww.plosone.org%2Fstatic%2Fpolicies.action%23sharing&data=05%7C01%7Cblevin%40emory.edu%7C5189c916b524474ad24108dbe47aaa4c%7Ce004fb9cb0a4424fbcd0322606d5df38%7C0%7C0%7C638354989737812245%7CUnknown%7CTWFpbGZsb3d8eyJWIjoiMC4wLjAwMDAiLCJQIjoiV2luMzIiLCJBTiI6Ik1haWwiLCJXVCI6Mn0%3D%7C3000%7C%7C%7C&sdata=G9RdDb9eD1wBEfY4tl9OhMQHPNGXFZ6txF8Ef%2FU9KIk%3D&reserved=0) requires authors to make all data underlying the findings described in their manuscript fully available without restriction, with rare exception (please refer to the Data Availability Statement in the manuscript PDF file). The data should be provided as part of the manuscript or its supporting information, or deposited to a public repository. For example, in addition to summary statistics, the data points behind means, medians and variance measures should be available. If there are restrictions on publicly sharing data—e.g. participant privacy or use of data from a third party—those must be specified.

Reviewer #1: Yes

Reviewer #2: No.

We don’t understand this assertion. We have made all the all data underlying the findings in their manuscript fully available. Please let us know.

Reviewer #3: Yes

4. Is the manuscript presented in an intelligible fashion and written in standard English?

PLOS ONE does not copyedit accepted manuscripts, so the language in submitted articles must be clear, correct, and unambiguous. Any typographical or grammatical errors should be corrected at revision, so please note any specific errors here.

Reviewer #1: Yes

Reviewer #2: No

Presumably the reason for this reviewer questions whether this manuscript is presented in an intelligible fashion and written in standard English is noted this reviewer’s comments to us. We have addressed all of these issues.

Reviewer #3: Yes

5. Review Comments to the Author

Please use the space provided to explain your answers to the questions above. You may also include additional comments for the author, including concerns about dual publication, research ethics, or publication ethics. (Please upload your review as an attachment if it exceeds 20,000 characters)

Reviewer #1: The authors describe a model to predict how the phage resistome of Lactococcus lactis drives phage evolution in allowing them to overcome abortive infection systems among other challenges to proliferation. The manuscript is well written, clearly organised and well executed. The approach is thorough and is well articulated. Some minor comments below are suggested to improve the manuscript.

Thank you for your flattering comments and appreciation for our manuscript.

1. In the abstract, the authors should mention which genus of phages are being used and what Abi systems are present in the host that will be discussed in the manuscript.

We have now done so.

Thanks for pointing this out. We added the taxonomic classification that corresponds to each phage. This is genus Skunavirus (family Siphoviridae) for phage p2 and family Siphoviridae for phage P335. We consider it important to maintain the traditional (former) nomenclature that refers to phage p2 as part of group 936 and P335 as part of group P335, since most previous studies with Lactococcus phages were performed using that nomenclature, and still useful for a wide audience. The International Committee on Taxonomy of Viruses (ICTV), especially the subbranch related to classifying bacterial viruses, has made important changes to the taxonomy of bacterial viruses in the last couple of years, and keeping both systems is useful to the reader during this process of changes. To our knowledge, there is no classification of taxa below the 'Genus' rank for the p2 virus, and below the 'Family' rank for the P335 virus.

2. The 936 group of phages have been reclassified as the Skunavirus genus. This should be updated in the manuscript.

Thank you, we have added Skunavirus to describe group 936.

3. The motivation for focusing on AbiZ is not clear. It's perfect to do so but needs explanation to the reader.
Thank you for pointing this out. On lines 67-69 (Introduction section) we tried to remark on the why of using AbiZ for evaluating the abortive infection dynamics in L. lactis in this study. We tried to highlight that AbiZ was used in this experimental work because this system conducts premature cell lysis during phage infection, and it is one of the more understood Abi mechanisms in *L . lactis*. Abortive systems in general have different mechanisms to produce cell suicide, and AbiZ is perhaps the most understood of all of them because of the abrupt and premature release of holins/lysins during the viral infection cycle. To make it clearer, we added the next to the paragraph: “and one of the most understood Abi mechanisms of *L. Lactis*.”

4. I cannot comment on the mathematical model but the approach seems rational and well considered.

Of course we agree.

5. The mutation rate is notably higher in p2 than P335. This difference is perhaps worth an explanation in the discussion.

As recommended, we have mentioned this difference in mutation rate in the Discussion.

It is important to mention that viral mutation rates can vary from host cells even when using the same virus. In this case, we were using two different virus-host systems. Even when both viruses, p2 and P335, are classified into the same family (*Siphoviridae*), they are sufficiently distant at phylogenetic level to be considered not only a different species but also to be classified in different viral taxon above the basic range of species. This, of course, has enormous implications in the biology of phage during the process of replication in the host cell, including the rate of mutations that can be generated and fixed in the viral population after each round of replication. However, both viruses, p2 and P335, are sensitive to the AbiZ, and this makes this story more interesting.

Reviewer #2: line 16: maybe revised
Background information is excessive, can be trimmed.

We disagree with this reviewer. We would like this manuscript to be read and understood by a broad audience, and not just specialists working on abortive infection and *Lactococcus lactis.* Towards this end, we consider it important to provide adequate background.

Material & Methods - should describe the research methods and study design more clearly
Results - the key findings of the study ought to be presented in a succinct fashion, generalities should be avoided.

We have gone through the materials and methods and have tried to present them in way that they will be read and understood by any reader interested in repeating these experiments.

The key findings of this report are summarized succinctly without generalities on lines 63-73.

Line 17-23: too much background information for abstract. the sentence " to combat this.......... and CRISPR-Cas" could be removed.

We disagree. Indeed, the first direct demonstration of CRISPR-Cas mediated immunity to phage infection was a study from investigators working in the dairy industry. {Barrangou, 2007 #20}. We believe it is useful to remind the readers of this, as many may not know or appreciate this. Moreover, we believe it’s critical for the readers to know the distinction between phage resistance due to receptor site modification, when the bacteria are refractory to the phage, and, immunity when the phage infects the bacteria and is lost, as is the case of Abi, restriction-modification and Crispr-Cas. Our manuscript makes this point and illustrates the difference between resistance and immunity.

line 24: species name abbreviated

We have modified the abstract to first introduce the full name of the bacterial species *Lactococcus lactis*.

Introduction:
Line 39: I suggest rewording

How does this reviewer want us to reword this? We believe it is clear as it is now written.

line 49-50: rephrase

How does this reviewer want us to rephrase this? We believe it clear as it is now written.

Line 51-52: what do you want to say here? kindly explain

We believe what we are saying is clear, but if the editor feels it’s not, we can modify.

line 55-56: rephrase

How does this reviewer want us to rephrase this? We believe it clear as it is now written.

line 57-58: what do the authors want to say here? The concept of population dynamics of Abi explained. line 55-59 should be rephrased

How does this reviewer want us to rephrase this? We believe it clear as it is now written.

line 63-67: confusing and ambiguous

We have reread this section and don’t believe it is confusing.

line 69-73: Why are the study findings discussed at the end of introduction section, instead of outlining the objectives of the study??

We believe where the objectives of a study are presented is arbitrary, as long as they proceed the results. The objectives of this study are presented in the abstract and the introduction.

Results:
line 75: statistical analysis is missing.

Where they are needed, as in Figure 4 standard deviations are included.

Avoid using general statements, merely describing an increase or decrease does not provide clarity, quantitative bent of your results would better convey the message.

The line graphs used to describe the population dynamics illustrate the direction and rates of change in the densities of the bacteria and phage are available for the reader. In the text we use words like increase or decrease to guide the reader about what they should be considering.

line 82: can you provide the quantitative data for this.

These data are presented in the figures.

What is meant by proliferate exponentially?

Populations of bacteria can grow, proliferate, exponentially.

Did you calculate growth rate etc.?

Yes, we did many times for the Abi+ and Abi- bacteria. Indeed, we have a sophisticated machine for this, a Bioscreen.

What do you mean by and how did you calculate the nutrient-limited densities?

We have modified this to say the bacteria consume the resource, and their populations grow exponentially until the nutrients needed for replication are depleted and reach their maximum density. We cited two articles about this: Stewart and Levin (1973) and Levin et al (1977), in the model we explain this.

line 86-87: ambiguous statements, provide quantitative data. How did you evaluate this?

The data are in Figure 1C and we are just describing these results.

Line 91-93: rephrase the sentences. What is meant by "as estimated"?

We have modified this to “**… the number of plaques formed”.**

line 94-98: Increases and decreases must be explained considering statistical differences. General result statements may not be helpful. Provide the quantitative data wherever is possible

We are describing results the reader can see from the figure.

Line 100: rephrase. What do you want to say here?

We believe what we said is clear as it is written.

line 129-133: ambiguous... Abi- or (Abi-) are the abbreviations used or N and Nr

We defined the variable N and Nr in Table 1.

Line 151-156: confusing. ? Can the equations be better explained here if that is the intent
We believe these equations are adequately explained and will be understood by readers who are all familiar with mathematical models of the population dynamics of bacteria and phage, whether they work with mathematical models or not.

Line 159-160: any reference for this?

It’s not clear what this review wants. The references are to the model which follows the noted lines.

Line 173: what do you mean by exactly by densities of bacteria and density of phages?

We have modified this line to include colony forming units (CFU) and plaque forming units (PFU) per mL for the densities.

Line 183-184: Rephrase

We don’t see what’s wrong with what we say in these lines.

line 207-212: Not comprehensible.

Some explanation would be helpful as to why this reviewer is having trouble understanding these passages.

Line 215-216: how did you evaluate this?

We are simply describing the result presented in this figure and table.

line 316: a critical and concise analysis of your results in the light of previously published literature is missing.

We believe the format and substance of this Discussion is consistent with the Discussions of virtually all studies of the population biology and evolution of bacteria and bacteriophage.

We are opening with a restatement of motivation for this study and a brief description of what we have done. In the paragraphs that follow we describe the results of this study and our interpretation of these results and their implications. We end with a consideration of the limitations of this study and encourage additional research addressing the population and evolutionary biology of abortive infection.

line 317: follow the same formatting throughout the manuscript

It’s not clear to us what this reviewer is getting at. Please be specific.

line 321-322: The mathematical model does not appear to account the evolution of envelope resistance? Clarify

We disagree. The mathematical model does account for the evolution of envelope resistance. It includes a population that is resistant to the phage and explores the conditions under which this resistance will evolve.

line 351-354: rephrase the entire sentence

We don’t know what this reviewer wants with this revision. Leaky resistance was defined in the previous sentence.

Line 389: CFU/ml?

We have fixed it.

Line 391-392: reason and ref?

We have added a reference to the reasons we use this media.

Line 395: bacterial name should be in italics throughout the manuscript

Thank you**.** We italicized the *L. lactis* here.

Line 396: full name?

We don’t believe Rodolphe Barrangou has any additional names.

line 402: ? define Abi escape.

We have modified this passage to indicate that Abi escape mutants are phages that can replicate on bacteria expressing Abi.

line 431-432: rephrase

Please expand. We don’t see what’s wrong with this passage.

line 435: check?

We have rewritten this passage.

… estimated with a fluctuation experiment with 10 independent cultures of 1E9 *L. lactis* were mixed with 1E9 phage in 3 mL soft agar. The number of colonies produced were estimated and rate of mutation to phage resistance calculated with the median method described in (38).

line 439: seems incomplete. short term experiment for?'

Short-term was modified to “Short-term bacterial growth and phage infection experiments”.

Line 444: how?

We changed “measured every hour” to “Samples were taken every hour and the phage and bacterial densities were estimated by serial dilution and plating”.

Line 513-514: how the densities were measured?

We added “Final densities of bacteria and phage were estimated by serial dilution and plating every 24 hours”.

Lin3 519: deg centigrade format is not correct

It was correct as written.

Reviewer #3: The authors embark to elucidate the conditions under which abortive infection confers protection to Lactococcus lactis against phages. They developed and numerically analysed a mathematical model of the population dynamics and interactions of bacteria harbouring abortive infection and phages, followed by an experimental validation. Generally, the article is well written and clear. However, a few things require some attention.

1. The justification for the mathematical model's purpose, which is ‘to provide a framework for a comprehensive consideration of the population dynamics of phage and bacteria with abortive infection’, appears to lack sufficient support.

The justification of the model is our addition of the escape mutant phage. This was not previously seen in the study done by Berryhill et al. 2023. The model allowed us to make predictions that were tested with experiments about this *L. lactis* abortive infection system. To our knowledge this is the first mass-action model for *L. lactis*, Abi, and Abi escape mutant phages.

The anticipation of resistance development, a foreseeable outcome based on existing knowledge, diminishes the model's scientific significance considerably. The authors should find a way to justify the importance of the mathematical model.

We disagree with the suggestion that the development of resistance to the infecting phage is a foreseeable outcome. Although in a previous jointly theoretical and experimental study of the population and evolutionary dynamics of abortive infection mediated by retrons {Berryhill, 2023 #21}, we did observe the evolution of envelope resistance and included it in the model used in that study, envelope resistance was not observed in studies of the population and evolutionary dynamics of CRISPR-Cas – mediated immunity in *Streptococcus thermophilus* {Levin, 2013 #22}. Moreover, for some phage, bacteria like *Staphylococcus aureus are* unable to generate viable resistant mutants {Berryhill, 2021 #4}. A major result of this study was to establish the population dynamics of *L. lactis* and Abi with its phages; envelope resistance was a key finding for these dynamics and the model we thus constructed specifically.

2. The mathematical model lacks some biologically relevant parameters like phage infection and replication rates in both ABI+ and ABI bacteria. Is there a reason why the authors did not include these parameters?

We disagree, the model includes the phage infection and replication rates in both ABI+ and ABI (ABI negative) phage. The difference in these two types of bacteria is the likelihood of abortive infection, the parameter q.

6. PLOS authors have the option to publish the peer review history of their article ([what does this mean?](https://nam11.safelinks.protection.outlook.com/?url=https%3A%2F%2Fjournals.plos.org%2Fplosone%2Fs%2Feditorial-and-peer-review-process%23loc-peer-review-history&data=05%7C01%7Cblevin%40emory.edu%7C5189c916b524474ad24108dbe47aaa4c%7Ce004fb9cb0a4424fbcd0322606d5df38%7C0%7C0%7C638354989737812245%7CUnknown%7CTWFpbGZsb3d8eyJWIjoiMC4wLjAwMDAiLCJQIjoiV2luMzIiLCJBTiI6Ik1haWwiLCJXVCI6Mn0%3D%7C3000%7C%7C%7C&sdata=SjyH038pzt4ARqbpIQaPyMCd1ehG3WHJqaSLNDCmgGw%3D&reserved=0)). If published, this will include your full peer review and any attached files.

If you choose “no”, your identity will remain anonymous but your review may still be made public.

**Do you want your identity to be public for this peer review?** For information about this choice, including consent withdrawal, please see our [Privacy Policy](https://nam11.safelinks.protection.outlook.com/?url=https%3A%2F%2Fwww.plos.org%2Fprivacy-policy&data=05%7C01%7Cblevin%40emory.edu%7C5189c916b524474ad24108dbe47aaa4c%7Ce004fb9cb0a4424fbcd0322606d5df38%7C0%7C0%7C638354989737812245%7CUnknown%7CTWFpbGZsb3d8eyJWIjoiMC4wLjAwMDAiLCJQIjoiV2luMzIiLCJBTiI6Ik1haWwiLCJXVCI6Mn0%3D%7C3000%7C%7C%7C&sdata=T4ezsaZ3PJG8Wu6ezOth4klhd58m7uho3k52L8USp%2BY%3D&reserved=0).

Reviewer #1: No

Reviewer #2: No

Reviewer #3: No

[NOTE: If reviewer comments were submitted as an attachment file, they will be attached to this email and accessible via the submission site. Please log into your account, locate the manuscript record, and check for the action link "View Attachments". If this link does not appear, there are no attachment files.]

While revising your submission, please upload your figure files to the Preflight Analysis and Conversion Engine (PACE) digital diagnostic tool, [https://pacev2.apexcovantage.com/](https://nam11.safelinks.protection.outlook.com/?url=https%3A%2F%2Fpacev2.apexcovantage.com%2F&data=05%7C01%7Cblevin%40emory.edu%7C5189c916b524474ad24108dbe47aaa4c%7Ce004fb9cb0a4424fbcd0322606d5df38%7C0%7C0%7C638354989737812245%7CUnknown%7CTWFpbGZsb3d8eyJWIjoiMC4wLjAwMDAiLCJQIjoiV2luMzIiLCJBTiI6Ik1haWwiLCJXVCI6Mn0%3D%7C3000%7C%7C%7C&sdata=4egJvjRuVws2NWZjFbATPQO5%2BA29HeKU6dwqudUQ9jo%3D&reserved=0). PACE helps ensure that figures meet PLOS requirements. To use PACE, you must first register as a user. Registration is free. Then, login and navigate to the UPLOAD tab, where you will find detailed instructions on how to use the tool. If you encounter any issues or have any questions when using PACE, please email PLOS at [figures@plos.org](mailto:figures@plos.org). Please note that Supporting Information files do not need this step.
